# The Senses of Agency and Ownership: A Review

**DOI:** 10.3389/fpsyg.2018.00535

**Published:** 2018-04-16

**Authors:** Niclas Braun, Stefan Debener, Nadine Spychala, Edith Bongartz, Peter Sörös, Helge H. O. Müller, Alexandra Philipsen

**Affiliations:** ^1^Department of Psychiatry and Psychotherapy, University of Bonn, Bonn, Germany; ^2^Medical Campus University of Oldenburg, School of Medicine and Health Sciences, Psychiatry and Psychotherapy, Oldenburg, Germany; ^3^Neuropsychology Lab, Department of Psychology, University of Oldenburg, Oldenburg, Germany

**Keywords:** virtual reality therapy, asomatognosia, alien hand syndrome, sense of ownership, sense of agency, rubber hand illusion, phenomenal transparency, limb-ownership

## Abstract

Usually, we do not question that we possess a body and act upon the world. This pre-reflective awareness of being a bodily and agentive self can, however, be disrupted by different clinical conditions. Whereas sense of ownership (SoO) describes the feeling of mineness toward one’s own body parts, feelings or thoughts, sense of agency (SoA) refers to the experience of initiating and controlling an action. Although SoA and SoO naturally coincide, both experiences can also be made in isolation. By using many different experimental paradigms, both experiences have been extensively studied over the last years. This review introduces both concepts, with a special focus also onto their interplay. First, current experimental paradigms, results and neurocognitive theories about both concepts will be presented and then their clinical and therapeutic relevance is discussed.

## Introduction

We usually take as granted that we possess a body and that we are agents, acting upon the world. Sense of ownership (SoO) describes the feeling of mineness that we perceive toward our body parts, feelings or thoughts (Gallagher, 2000), whereas sense of agency (SoA) refers to the experience of initiating and controlling an action (Moore and Fletcher, 2012). Although in most daily life situations we do not reflect upon such experiences, both experiences play a fundamental role in our life. In fact, both experiences are thought to play an important (Blanke and Metzinger, 2009), if not indispensable (Gallagher, 2000) role in any self-experience. Accordingly, over the last years, many empirical investigations on SoO and SoA have been carried out and their clinical and philosophical relevance has been discussed. This review introduces both concepts and discusses recent evidence addressing their interplay, enabling mechanisms and clinical relevance. The review is structured as follows: First, the different paradigms, findings and theories of SoO and SoA will be introduced. Next, the interplay between both phenomenal experiences as well as their clinical and therapeutic relevance will be discussed.

## Sense of Ownership

As mentioned above, SoO describes the feeling of mineness that we experience toward our body parts, feelings or thoughts. It is the feeling that is described in statements such as “*This is* ‘*my*’ *hand*,” “*It is* ‘*me*’ *who is thinking this thought*” or “‘*I*’ *am the one who is having this feeling*.” As such, although often experienced at the fringe of consciousness (De Vignemont, 2011), SoO has a complex and non-unitary phenomenal structure (Tsakiris, 2010, 2016). Most of the research conducted so far has focused on the sense of body ownership. Therefore, this type of SoO will be the major, but not exclusive focus of this review. Where necessary, a distinction will be made between limb-ownership (also called limb-identification) and body-ownership (also called self-identification). Whereas body-ownership refers to any “*globalized form of identification with the body as a whole*” (Blanke and Metzinger, 2009, p. 8), limb-ownership refers to SoO toward some specific body part. In the next subsections, experimental paradigms, phenomenological and neurocognitive theories as well as the neuroanatomical substrates of SoO will be summarized.

### Experimental Investigation

Over the last two decades, several experimental paradigms have been developed that allow a systematic manipulation of SoO. While some of these paradigms target limb-ownership, other paradigms concentrate on more global aspects of bodily self-awareness.

**FIGURE 1:**
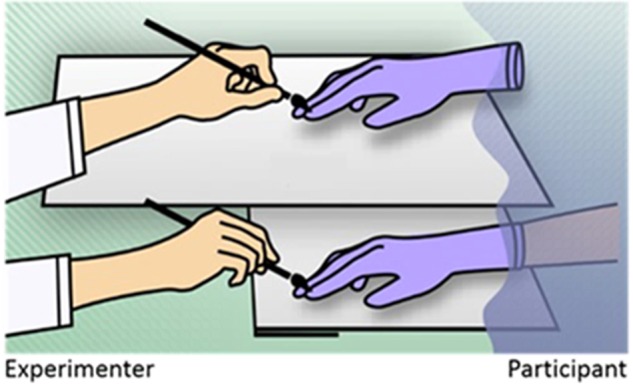
The classical rubber hand illusion. The participant’s hidden hand **(lower shelf)** and rubber hand **(upper shelf)** are synchronously stroked by the experimenter. In most participants this induces an illusory SoO over the rubber hand.

As regards limb-ownership, the predominant paradigm is the rubber hand illusion (RHI; Botvinick and Cohen, 1998). In its original setting, an artificial hand is placed visibly, and in an anatomically plausible position, in front of a participant, while the participant’s own hand is hidden from view (see **Figure 1**). The experimenter then repeatedly strokes both the artificial hand and the real hand in synchrony. In most participants this induces an illusory SoO over the artificial hand. Likewise, if the participants then are asked to blindly localize the position where they experience their hand to be, they tend to mislocalize their real hand’s position toward the artificial hand, an observation that has been named proprioceptive drift. Also, applying a potentially painful manipulation to the artificial hand can produce a strong physiological fear response, regardless of whether the artificial hand is actually threatened (Armel and Ramachandran, 2003; Braun et al., 2016) or only approached by the threatening object (Ehrsson et al., 2007; Guterstam et al., 2011). This physiological result has been interpreted as implicit evidence for a successful embodiment of the artificial hand (Armel and Ramachandran, 2003; Ehrsson et al., 2007; Alimardani et al., 2013; Braun et al., 2016).

Since its original conception, many different variants of the RHI have been developed (for a review, see Kilteni et al., 2015). While the first RHI-studies exclusively induced the RHI by visuotactile stimulation, newer studies also used other crossmodal stimulation combinations. Ehrsson et al. (2005) for instance developed a somatic RHI variant, which only requires proprioceptive and tactile stimulation, but no visual stimulation. To induce this illusion, the experimenter moves the blindfolded participant’s left index finger such that it strokes the index finger knuckle of an artificial hand and synchronously he strokes the participant’s real right index finger’s knuckle (Ehrsson et al., 2005). Also different active RHI variants have been developed where an artificial or virtual hand is not touched, but moved in synchrony with the participant’s real hand movements (Slater et al., 2009; Walsh et al., 2011; Kalckert and Ehrsson, 2012, 2014a,b; Romano et al., 2014) or with only imagined hand movements (Slater et al., 2009; Braun et al., 2016) (details in Section “Interplay between SoA and SoO”). All these studies show that visuotactile stimulation is not the only possibility to induce illusory SoO over an artificial hand.

Instead of inducing SoO over a mechanical hand, more recent studies have also experimented with different “virtual hand illusion” (VHI) settings where a virtual hand becomes presented on a screen or in an immersive 3D virtual space (Slater et al., 2008, 2009; Sanchez-Vives et al., 2010; Kilteni et al., 2012; Ma and Hommel, 2013; Pichiorri et al., 2015; Ma et al., 2017). These VHI studies not only replicate the general finding that SoO can be induced over an artificial (virtual) hand, but they also allow for more flexible manipulations that cannot be done in a classical RHI setting. For instance, a virtual hand shape could be dynamically changed, or the virtual hand could be freely moved (Ma et al., 2017). With the ongoing maturation of VR technology, many new insights upon SoO (and SoA) may thus be expected by this new technique.

Besides the RHI and its alterations, other *body transfer illusions* (BTI) have also been reported. Prominent paradigms are for instance the Butcher‘s tongue illusion where illusory SoO is perceived for an artificial out-of-body tongue (Michel et al., 2014); the enfacement illusion where one’s own mental face representation assimilates to another person’s or virtual face (Tsakiris, 2008; Sforza et al., 2010; Beck et al., 2015; Ma et al., 2017); and the rubber foot illusion where illusory SoO is perceived over an artificial foot (Crea et al., 2015). Significantly fewer studies, however, have been conducted for these BTI studies as compared to the tremendous RHI-research. Nonetheless, the so far conducted research suggests that many of the derived findings from RHI-research (see How Does SoO Emerge?), also translate to other body parts.

Other studies focus on body-ownership rather than limb-ownership. From a philosophical perspective, these *out of body illusion (OBI)* studies are particularly interesting, since here not only the experience of one body part, but the bodily self as a whole is concerned (Metzinger, 2007b; Blanke and Metzinger, 2009). Among the first OBI studies were Lenggenhager et al.’s (2007) study and Ehrsson’s (2007) study. In Lenggenhager et al.’s (2007) study, participants got equipped with a head-mounted display by which they could observe their own back, as if they were sitting behind themselves. The experimenter then stroked the participants’ backs while the participants synchronously observed this stroking in the video. As a result, the participants partly identified the virtual body as their own body and spatially mislocalized themselves toward the virtual body position. In Ehrsson’s (2007) study, a subtly different setting was used. Also here, the participants wore a head-mounted display by which they observed their own back from behind. However, this time the experimenter stroked the participants’ chest with one stick while moving another stick just underneath the camera position, as if it were stroking another (virtual) body. As a result, many participants reported an illusory self-displacement, in that they experienced themselves sitting behind their own bodies, watching their own backs. Since these two seminal studies, several further OBI studies have been carried out using similar setups (Petkova and Ehrsson, 2008; Aspell et al., 2009; Ionta et al., 2011; Pfeiffer et al., 2013; Salomon et al., 2013). Taken together, one important outcome is that at least three different types of bodily self-experience can be experimentally dissociated: self-identification (i.e., global body-ownership), self-location (i.e., the experience of where ‘I’ situate myself in space) and first person-perspective (i.e., the experience of the position from where ‘I’ perceive the world) (Blanke, 2012). This shows that, although on the phenomenal level an experience of a unitary and coherent self may arise, this self-experience is in fact not ontologically substantial (Metzinger, 2007a).

### How Does SoO Emerge?

The question of how SoO emerges has been addressed by many disciplines, among them phenomenology, philosophy of mind and cognitive neuroscience (for reviews, see Tsakiris, 2010, 2016; De Vignemont, 2011). Following the taxonomy put forward by Tsakiris (2010), most neurocognitive theories can be placed in a continuum somewhere between bottom–up and top–down accounts. Whereas bottom–up accounts assume that SoO mainly depends on multisensory integration and only marginally on internal body maps, top–down accounts assume a much stronger involvement of internal body maps (Tsakiris, 2010).

#### The Bayesian Perceptual Learning Theory

The perhaps strongest bottom–up account has been posited by Armel’s and Ramachandran’s (2003) Bayesian perceptual learning theory in arguing that visuotactile correlation is both necessary and sufficient to induce the RHI. That is, according to this theory, any object can be experienced as part of one’s body, as long as the visual and tactile information coming from this object spatiotemporally correlates, and thus can be interpreted to occur from one common event. To substantiate their theory, Armel and Ramachandran (2003) conducted an RHI-like study, in which the rubber hand or only a table without a rubber hand was either stroked in synchrony or in asynchrony with the stroking of the participant’s hidden real hand. Findings revealed, that participants reported some SoO toward the table, but only if the stroking was done synchronously (Armel and Ramachandran, 2003).

While Armel’s and Ramachandran’s (2003) study contradicts with other RHI studies that find no evidence for a SoO-inducibility over non-hand objects (Tsakiris and Haggard, 2005; Haans et al., 2008; Tsakiris, 2008, 2010; Tsakiris et al., 2010a), Hohwy and Paton (2010) partly replicate Armel’s and Ramachandran’s study. Using a head-mounted display, they investigated, whether a touch illusion for non-hand objects can be induced. To this end, they first induced a VHI in their participants by synchronously tapping the participant’s real forearm and a virtually presented forearm. The virtual forearm was thereby presented at that virtual space position that corresponded to the participant’s expected forearm position in real space. Next, after 30 s VHI-induction, the virtual forearm suddenly disappeared and instead a virtual cardboard box appeared at the same position. As before with the virtual forearm, the virtual cardboard box was tapped in synchrony with the still continuing tapping of the participant’s real hand. As a result, some participants reported a touch illusion for the non-hand box. Interestingly, however, as revealed by a control condition, this illusion was not invocable if the preceding VHI induction was omitted. In trying to explain their results, Hohwy and Paton (2010) suggest a similar, although weaker, bottom up approach as Armel and Ramachandran (2003). According to their approach, although transtemporally stable internal body maps exist, SoO mainly depends on Bayesian inference of afferent input. That is, if it comes to a conflict between the current afferent input and the internal body maps, the internal body maps become situationally adapted, or if needed, “explained away” (Hohwy and Paton, 2010).

#### The Neurocognitive Model of SoO

The neurocognitive model (NCM) of SoO (Tsakiris, 2010) is a typical top-down account of SoO. According to the NCM, SoO results from integrating different information sources into pre-existing, internal body maps. More specificially, the NCM proposes a three-level comparator mechanism for inferring SoO. The first comparator contrasts the visual appearance of an observed object against a pre-existing, transtemporally stable body model. This body model entails anatomical and structural descriptions of the person’s body and if the observed object bears enough perceptual similarity to this model, the second comparator is consulted. This second comparator then contrasts the current body schema state (i.e., the body’s currently estimated postural configuration) against the anatomical, structural and postural properties of the observed object. If there is enough perceptual similarity between the observed object’s posture and the current body schema state, the third comparator comes into play. This comparator matches the different sensory information about the observed object (e.g., vision of touch vs. felt touch) and if it matches, SoO for observed objects ultimately arises.

While it may be questioned whether the brain literally implements such a three-level comparator mechanism for inferring SoO, the NCM’s main assertion that there are at least some internal body maps structuring our somatosensory input is now well supported by empirical research. Evidence for a top–down modulation of SoO comes for instance from a range of RHI studies, indicating that besides intermodal synchrony anatomical, spatial, postural and textural constraints have to be fulfilled as well.

As regards anatomical constraints, one typical finding is that a full-blown SoO experience is typically only inducible for body-shaped objects, but not for non-body–shaped objects, such as a wooden block (Haans et al., 2008; Tsakiris, 2008, 2010; Tsakiris et al., 2010a; Tsakiris and Haggard, 2005). One exception here is the already mentioned study by Armel and Ramachandran (2003) which also reported SoO toward a table. However, even here the reported SoO level was weaker for the table stroke condition as for the classical rubber hand condition.

A spatial constraint is that the strength of the RHI depends on the distance between the rubber hand and the participant’s real hand (Armel and Ramachandran, 2003; Lloyd, 2007; Zopf et al., 2010; Preston, 2013; Kalckert and Ehrsson, 2014b). Agreement exists in that the greater the distance is, the weaker is usually the RHI, regardless of whether a horizontal (Armel and Ramachandran, 2003; Lloyd, 2007; Zopf et al., 2010; Preston, 2013) or vertical RHI setting (Kalckert and Ehrsson, 2014b) is used. Controversy exists, however, as to how far both hands may be apart from each other until the illusion completely decays. For the horizontal RHI setting, Lloyd (2007) found that the illusion decays for a 27 cm distance, whereas other research groups even reported successful RHI inductions for 45 cm (Zopf et al., 2010) and 91 cm distances (Armel and Ramachandran, 2003). For the vertical RHI setting, Kalckert and Ehrsson (2014b) reported a maximum RHI inductibility distance of 27 cm in case of the classical RHI and a maximum RHI inducibility distance of 12 cm in case of the active RHI.

A postural constraint is that the artificial hand needs to be anatomically aligned to the participants real hand (Ehrsson et al., 2004; Tsakiris and Haggard, 2005; Costantini and Haggard, 2007; Kalckert and Ehrsson, 2012; Braun et al., 2014). If the artificial hand becomes for instance rotated by 180 degree, i.e., becomes placed in an anatomically implausible position for the participant, the illusion typically diminishes (Tsakiris and Haggard, 2005; Kalckert and Ehrsson, 2012; Braun et al., 2016, 2014).

A textural constraint is that if the rubber hand skin texture looks natural, the RHI is experienced more strongly than if it looks unnatural (Haans et al., 2008) or does not match with the own skin color (Farmer et al., 2012; Lira et al., 2017). Lira et al. (2017), for instance, recruited white participants and compared the inducibility of a RHI with a white and with a black rubber hand. What they found was that for the black rubber hand condition, there was a longer RHI induction time, lower SoO level and smaller proprioceptive drift than for the white rubber hand condition (Lira et al., 2017).

In summary, while there is currently strong agreement that there are at least some internal body maps structuring our somatosensory input, the question remains as to how strong this top–down modulation may be.

#### The Self-Model Theory of Subjectivity

An interesting phenomenological note on explaining SoO can be found in Thomas Metzinger’s self-model theory of subjectivity (SMT) (Metzinger, 2003, 2007b). In brief, Metzinger’s assumption is that for a conscious self-representation to be experienced as mine, it has to become transparent. A mental representation is thereby said to be transparent if only its content properties become introspectively accessible, but not its vehicle properties (Metzinger, 2007a). In other words, transparency occurs in the moment when the representational character of a representation’s phenomenal content is not co-represented anymore. If this happens, so Metzinger’s proposal, the subject of experience “directly looks through” its own mental representation, as if it was in “direct and immediate contact” with the representation’s content (Metzinger, 2007a, p. 236). As a consequence, the subject of experience perceives this representational content as real, and, if it is self-representational, as mine.

For a concrete example of a how limb-ownership may arise according to the SMT, consider Ramachandran’s mirror box experiments with phantom limb patients (Ramachandran and Rogers-Ramachandran, 1996; Metzinger, 2007a; Ramachandran and Altschuler, 2009). In these experiments, the patients’ healthy limb is first placed in front of a mirror. Next, the mirror is placed in such a way that from the patients’ view, the healthy limb’s mirror reflection is superimposed on the place where the amputated limb would have been. When patients are then asked to conduct movements with their healthy limb and attend to the mirror reflection, many report a strong increase in the vividness of their phantom limb (Ramachandran and Rogers-Ramachandran, 1996; Metzinger, 2007a; Ramachandran and Altschuler, 2009). That is, for the duration of the experiment, they experience their amputated/phantom limb as though present again, as if it is physically there and voluntarily movable again. The SMT explanation here would be that, for the duration of the experiment, the patients phenomenally “forget” about the representational character of their mental “mirror-limb”–representation and therefore interpret the representation’s content as real, and since this content is self-representational, also as mine.

If Metzinger’s view holds, then his theory should explain why under some clinical conditions, patients experience reduced, or even complete absence of, thought-ownership (e.g., thought insertions), limb-ownership (e.g., asomatognosia) or body-ownership (e.g., depersonalization). A first intuitive explanation would be that in these patients some of their self-representations are too opaque, that is, they still co-represent their representational character. As a consequence, these representations are no longer experienced as immediately given anymore, but instead as distant and alien (Metzinger, 2003).

### Neuronal Correlates

Several studies have investigated the neuronal correlates of SoO (for reviews, see Tsakiris, 2010, 2016; Blanke, 2012). Like most behavioral SoO studies, these neuroimaging studies typically rely on RHI or VHI paradigms. Ehrsson et al. (2004) for instance developed a RHI variant where participants may lie within a functional magnetic resonance imaging scanner (fMRI) and where control conditions like in the classical RHI can be used. Besides fMRI studies, positron emission tomography (PET), electroencephalography (EEG) and lesion mapping studies have been carried out, as well as a few invasive neurophysiological recordings in monkeys.

As regards EEG studies, several studies have investigated ERP correlates as well as coherence correlates of illusory hand-ownership during the RHI (Peled et al., 2003; Kanayama et al., 2007, 2009; Zeller et al., 2015; Faivre et al., 2017; Rao and Kayser, 2017). The ERP studies thereby mainly focused on the somatosensory evoked potentials (SEPs) that result from brush-stroking the artificial hand. Comparing these SEPs between conditions inducing an RHI and different control conditions, relative SEP attenuations have been reported at around 55 ms over left frontal and right parietal electrodes (Zeller et al., 2015), at around 460 ms over frontal electrodes (Peled et al., 2003) and at around 330 ms over frontocentral electrodes (Rao and Kayser, 2017). Given the involvement of frontocentral and parietal electrodes, a contributory role of the premotor cortex (PMC) and intraparietal sulcus (IPS) in inducing hand-ownership is therefore suggested (Rao and Kayser, 2017). A contributory role of the parietal cortex was also found in two EEG coherence studies that consistently reported positive correlations between RHI vividness and interelectrode phase synchrony of the lower gamma band (40–50 Hz) over parietal scalp regions (Kanayama et al., 2007, 2009).

As regards fMRI and PET studies, bilateral PMC (Ehrsson et al., 2004; Petkova et al., 2011; Brozzoli et al., 2012; Gentile et al., 2013; Bekrater-Bodmann et al., 2014), IPS subregions (Ehrsson et al., 2004; Petkova and Ehrsson, 2008; Brozzoli et al., 2012; Gentile et al., 2013), extrastriate body area (Limanowski et al., 2014), the putamen (Petkova et al., 2011) and the insula (Tsakiris et al., 2007a; Limanowski et al., 2014) have been associated with experimentally induced SoO and/or hand-centered coordinate systems. For the PMC and IPS, it has been proposed that multimodal neurons – integrating visual and somatosensory information – may be responsible for encoding one’s own phenomenal corporeal space. Using single-cell recordings, such neurons have been repeatedly found in primates and are known to respond to stimuli applied to a limb as well as to visually approaching this limb (Hyvarinen and Poranen, 1974; Rizzolatti et al., 1981a,b; Graziano et al., 1994; Duhamel et al., 1998). Interestingly, in monkeys, such arm-centered multimodal IPS neurons became active during a RHI-like setting (Graziano et al., 2000; see also Blanke, 2012 for a critical discussion). An involvement of the insula in the SoO also appears plausible, given its functional role in affective self-awareness and interoceptive integration (Craig, 2009). In fact, several theoretical accounts have been recently put forward, arguing that interoception plays a key role in grounding the phenomenal self (for a review and critical discussion, see Tsakiris, 2016). Moreover, one voxelwise lesion-behavior mapping study exist that finds the right posterior insula to be lesioned in stroke patients suffering from somatoparaphrenia (see Limb-Specific Disruptions of SoO and SoA) (Baier and Karnath, 2008). In short, although several brain regions have been proposed to contribute to SoO, a consistent picture with respect to the exact involvements of the mentioned areas is still missing.

## Sense of Agency

As stated above, SoA is the phenomenal experience of initiating and controlling an action. It is the feeling of authorship that we refer to when we say sentences like “*I am the one who is in control of this car*” or “*It must have been me who just pressed this button*.” As such, SoA phenomenally distinguishes our own self-generated actions from those actions generated by others (David et al., 2008; Moore, 2016). While SoA is sometimes exclusively linked to mere motor control, it may also entail further intentional aspects ranging beyond our bodily boundaries (Gallagher, 2010, 2012). For instance, if I press a button (the motor aspect of my action), I usually do this for some purpose, like booking a flight (the intentional aspect of my action). In this wider sense, SoA is the phenomenal target property upon which many ethical and juridical concepts such as moral responsibility, free will and guilt are based on (Wegner, 2002; Moore, 2016; Haggard, 2017). In the following, a conceptual distinction between two different SoA levels will first be introduced and then some recent experimental paradigms and neurocognitive theories will briefly be reviewed.

### Different SoA Levels

As it is the case with SoO, SoA occupies a complex and non-unitary phenomenal structure. Several authors have argued for distinct SoA levels (Synofzik et al., 2008a; Jeannerod, 2009; Gallagher, 2012; Moore et al., 2012). One influential distinction comes from Synofzik et al. (2008a), who proposed a multifactorial two-step account. According to this theory, a ‘feeling of agency’ (FoA) level can be distinguished from a judgment of agency (JoA) level. The FoA level is described as being pre-reflective, low-level and non-conceptual. That is, it operates at the fringe of consciousness and occupies a rather ‘thin’ phenomenology.

The JoA level, by contrast, is characterized as a higher-order, belief-like process. Its phenomenology is more complex, may range beyond mere motor control and reflects a person’s judgment of being the author of an action. Besides motor information, it hinges on a post-hoc reconstruction of authorship, contextual knowledge and background beliefs (Synofzik et al., 2013; Moore, 2016). Although on a theoretical level, Synofzik et al.’s (2008a) account has widely received support, we are only aware of one empirical study that investigated the interplay between both levels and found evidence for a dissociation of the two levels (Moore et al., 2012). For simplicity, in the remainder of this review, SoA will be used as a superordinate term, unless either the FoA or JoA level is specifically addressed.

### Experimental Investigation

Many different experimental paradigms exist to study SoA. Following Moore’s (2016) taxonomy, these paradigms can be broadly separated into two categories: either they use implicit methods or explicit methods of assessing SoA. In the following, these measures will only be briefly described, since extensive reviews can be found elsewhere (De Vignemont and Fourneret, 2004; David et al., 2008; Moore and Obhi, 2012; Moore, 2016).

#### Implicit SoA Measures

**FIGURE 2:**
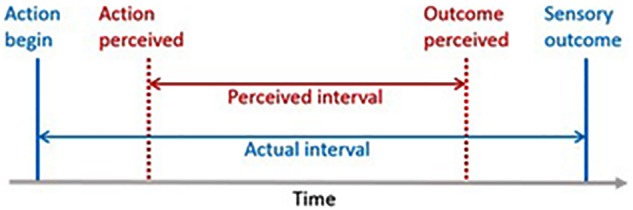
The intentional binding paradigm. Participants judge the time interval between a voluntary action (e.g., a button press) and its sensory outcome (e.g., an occuring sound). A typical outcome is that participants subjectively underestimate the actual time interval between action onset and sensory outcome (Moore and Obhi, 2012).

Implicit SoA measures assess some behavioral or neurophysiological correlate of voluntary action (Moore, 2016). Hence, in these paradigms the participants are not explicitly asked about their own agentic experience, but their experience is inferred from a measured correlate. Typically, but not exclusively, implicit SoA measures relate to the FoA level. Besides sensory attenuation paradigms (Blakemore et al., 1998), the most widely used implicit SoA measure is intentional binding (for a review, see Moore and Obhi, 2012). The intentional-binding effect refers to the subjective compression of time experienced between a voluntary action (e.g., a self-conducted button press) and its external sensory consequences (e.g., a sound played thereafter) (see **Figure 2**). A common finding is that the time interval is only underestimated when the action is voluntarily, but not when it is involuntarily (Haggard et al., 2002; Haggard and Clark, 2003) or passively conducted (Wohlschläger et al., 2003a; Engbert et al., 2008). Moreover, there is some evidence that intentional binding is stronger for self-generated than observed voluntary actions (Engbert et al., 2007, 2008). Given these findings, Moore and Obhi (2012) suggested that temporal binding results from an efference-based prediction mechanism that binds together an intention-to-act with the corresponding sensory outcome. While this view has become prevalent in the field, some authors have also challenged it. One important objection is that some studies could not find any differences between self-generated and observed voluntary actions (Wohlschläger et al., 2003a,b; Poonian and Cunnington, 2013) or could only trendwise replicate the above-mentioned effect (Braun et al., 2014). Moreover, some studies even observed temporal binding effects in the absence of voluntary actions (Buehner and Humphreys, 2009; Buehner, 2012). As a consequence, Buehner and Humphreys (2009) and Buehner (2012) suggested that causal inference in general, rather than intentionality or agentive inference, leads to temporal binding. Further studies are necessary to explore the neurofunctional underpinnings of the intentional binding effect and how well it can be attributed to SoA.

#### Explicit SoA Measures

In contrast to implicit SoA measures, explicit measures directly assess some aspect of the participants’ SoA experience (Moore, 2016). Often, this is achieved through subjective questionnaires where the participants judge their contribution to an experimental task or describe how vividly they experienced SoA during that task. Popular paradigms using explicit SoA measures are for instance Wegner et al.’s (2004) ‘helping hands’ experiment (see The Comparator Model), Wegner’s and Wheatey’s (1999) ‘I spy’ experiment (see The Retrospective Inference View) and Aarts et al.’s (2005) “wheel of fortune” experiment. In the latter experiment, participants had to move a gray square rapidly traversing a rectangular grid by holding the ‘s’-key, while at the same time, the computer independently moved another gray square with the same speed but into the opposite direction. The grid thereby consisted of eight white tiles. Next, participants were instructed to press the ‘enter’-key to stop the motion of their square. As soon the ‘enter’-key was pressed, a black square appeared on one of the grid’s white tiles and the participants were told that this black square either represented the location of their own gray square or the location of the computer’s gray square at the time point the ‘enter’-key was pressed. In fact, the black square’s position was pre-determined in most of the conditions. Next, the participants were asked to judge on a Likert scale how strongly they felt of having controlled the black square’s position. One interesting result was that subliminal or supraliminal priming of the black square’s position enhanced the participant’s reported SoA for stopping the square. This has been interpreted as evidence for the retrospective inference view (see The Retrospective Inference View). Another explicit way of assessing SoA are experiments where the participants may perform a motor task which they cannot directly observe (Moore, 2016). Instead they only see some motor feedback on a screen which depicts either their own movements or the movements of someone (e.g., the experimenter) or something (e.g., a computer) else. And the participants are then asked to judge whose movement is seen on the screen.

### How Does SoA Emerge?

#### The Comparator Model

**FIGURE 3:**
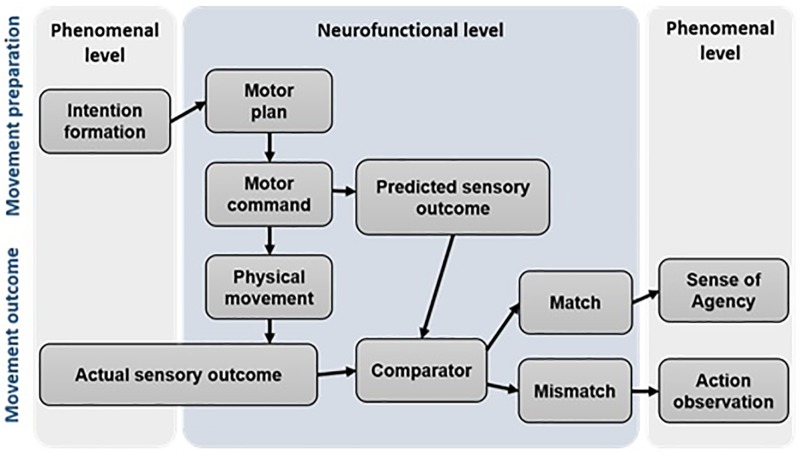
A simplified illustration of the comparator system. (Adapted from: David et al., 2008).

Although originally developed as a theory of motor control, the comparator model (CM) is also used today to explain the occurrence of SoA (Feinberg, 1978; Frith, 2005; David et al., 2008). According to this theory, the brain possesses an internal prediction model, which induces an efference copy whenever a new motor command is generated. If the efference copy matches the actual sensory input (reafference), the movement is perceived as self-caused and SoA arises. In case that efference copy and sensory input do not match, no SoA arises. A graphical illustration of the CM is given in **Figure 3**.

As a theory of motor control, the CM is nowadays well-supported by empirical studies (David et al., 2008; Synofzik et al., 2008a; Gallagher, 2010). As to how far the CM also captures the neurocognitive mechanisms underlying SoA is, however, less clear (David et al., 2008; Synofzik et al., 2008a; Moore, 2016). One often-raised critique is that the CM only considers sensorimotor processes relevant for SoA and neglects a contributory role of any other agency cues (Wegner and Wheatly, 1999; Synofzik et al., 2008a; Moore, 2016). In many situations, however, so the critique, other agency cues, provide a more reliable information source for inferring agency. Another related objection is that there is clinical and experimental evidence that SoA can be experienced in the absence of sensory reafference, and thus without a comparator mechanism (for critical discussions, see Synofzik et al., 2008a; Carruthers, 2012). Two clinical examples are phantom limb patients that experience their phantom limb to be voluntarily movable (Ramachandran and Hirstein, 1998) and deafferented patients that still report SoA for their only visually guided actions (Cole and Paillard, 1995). An experimental example is Wegner et al.’s (2004) “helping hand”-study where participants watched themselves in a mirror while another person stand directly behind them. The person behind extended and moved his or her arms in such a way that in the mirror the visual impression was generated that the participants themselves moved their arms. One interesting outcome was that if the participants became verbally informed about the next movements, they reported SoA for these movements, although they factually did not move (Wegner et al., 2004). As Carruthers (2012) works out, although the so far presented evidence against the CM necessitates considerable case-by-case modifications of the original CM (e.g., assuming an only simulated sensory reafference in phantom limb patients), it is not strong enough to falsify the CM.

#### The Retrospective Inference View

The retrospective inference view, or ‘theory of apparent mental causation’ (Wegner and Wheatly, 1999) approaches the question of how SoA arises from a quite different perspective than the CM. As clarified by Moore et al. (2012), Moore (2016) whereas the CM assumes motor prediction to be the major neuronal mechanism behind SoA, the retrospective inference view rejects such a strong involvement of the motor system in SoA. Instead, it introduces SoA as resulting from a ‘general-purpose inferential mechanism’ that infers the causal influence onto an observed action from the sensory input (Moore and Obhi, 2012). According to Wegner’s model, SoA occurs from retrospective inference and arises if (1) an intention precedes an observed action (priority), (2) the intention is compatible with this action (consistency) and (3) the intention is the most likely cause of this action (exclusivity) (Wegner and Wheatly, 1999; Moore and Obhi, 2012).

A few empirical studies provide some support for the retrospective inference view. One popular study is for instance the “I spy”-experiment by Wegner and Wheatly (1999). In this study, participants and an experimental confederate jointly controlled a computer mouse that could be freely moved over a series of pictures shown on the computer screen. For each trial, the participants and confederate thereby had the task to point with the cursor at one of the presented pictures after about 30–40 s and then to indicate on a Likert scale their level of action contribution in the current trial. One interesting outcome was that when the participants had been primed before with the name of the chosen picture, they were more likely to attribute the action to themselves, even when the picture was chosen by the confederate and not by the participant. Similar overestimations of self-agency have also been documented for other experiments (cf. the above-mentioned ‘wheel of fortune’ experiment and the ‘helping hands’ experiment) (for critical discussions, see Wegner, 2002; David et al., 2008; Carruthers, 2012, 2015).

An interesting philosophical implication that Wegner derives from his account is that SoA is illusory, in the sense that our “experience of consciously willing an action” is not causally involved in the generation of action (Wegner, 2002; p. 2). This interpretation and the retrospective inference view in general, has, however, been criticized from different directions for empirical and conceptual reasons (for critical discussions, see e.g., Carruthers, 2010; Walter, 2014).

#### The Multifactorial Weighting Model

The multifactorial weighting model (MWM) seeks to find a compromise between the CM and retrospective inference view (Synofzik et al., 2008a). Relying on the FoA vs. JoA distinction, this account suggests that SoA comes about by many different SoA cues, which become constantly weighted according to their reliability in a given situation. That is, the MWM does not deny a contributory role of the CM in many SoA situations, but it suggests that other SoA cues also contribute to the emergence of SoA. If a certain action, for instance, does not allow for a precise efference-reafference–comparison, the brain also takes into account other SoA cues. This appears to be particularly happening for JoA-situations where social and environmental cues provide a much more reliable information source than mere efference-reafference–comparisons. As an example, Synofzik et al. (2008a, p. 8) mention here the case of sitting alone in a room and witnessing an action: “*I may believe that I am the agent of the action, just because I take into account the fact that I am alone in the room*.” If the MWM is right, the question is how the brain assigns the weights to the different agency cues in a context-dependent manner. While in their initial paper, Synofzik et al. (2008a) remained rather vague upon this question, their current proposal is that the weighting of the different agency cues is realized by Bayesian cue integration (Synofzik et al., 2009, 2010; Moore and Fletcher, 2012; Vosgerau and Synofzik, 2012).

#### The Bayesian Cue Integration Theory

Although developed as a SoA theory on its own, the Bayesian cue integration theory (BCIT) by Moore and Fletcher (2012) provides an elegant mathematical theory of how the MWMs proposed multifactorial weighting of different agency cues might be realized. The BCIT’s background assumption is that the brain has access to many different agency cues (information channels), each providing their own estimates about the agentic origin of an event. These agency estimates, however, are highly noisy signals (i.e., have a high signal variance), so that for each agency cue there is estimate uncertainty. Consequently, the brain cannot simply rely on one agency cue, but needs to optimally aggregate all relevant information coming from its different agency cues. In order to do so, so Moore’s and Fletcher’s suggestion, the brain calculates an overall estimate out of all its agency cues, where the weight of each cue depends on its individual precision. More specifically, Moore’s and Fletcher’s proposal is that the brain applies a Maximum likelihood estimation (MLE) to all its agency cues and thereby derives an overall agency estimate, whose signal variance (noise) is much lower than the signal variances of any agency cue alone (Moore et al., 2009; Moore and Fletcher, 2012). Interestingly, MLE has not only been proven to provide a statistically optimal solution to the cue integration problem (Rohde et al., 2016), but there is also evidence from perceptual research that the brain actually often integrates its multisensory information in a MLE-like manner (for a review, see van Dam et al., 2014). Therefore, Moore et al. (2009, 2012) suggest that a MLE-like cue integration mechanism also serves as the basis for inferring agency. Another interesting aspect is that MLE operates data-driven and requires no prior knowledge about which agency estimates to expect. However, as Moore and Fletcher work out, such prior information can be easily integrated to the model by Bayesian priors (for more details, see Moore and Fletcher, 2012). Hence, three important advantages of the cue integration theory are (1) that it provides a parsimonious explanation of how different agency cues can be optimally integrated to one overall agency inference mechanism; (2) that it is flexible enough to integrate many different agency cues; and (3) that it allows to integrate prior knowledge (top–down constraints) into the model. The BCIT’s and MWM’s explanatory power and flexibility, brings, however, also some unresolved questions with it. No concrete assertions are for instance given by either model on how many agency cues actually exist. This makes it difficult to falsify these models (for a critical discussion, see Carruthers, 2012; but also see, Vosgerau and Synofzik, 2012 for a reply).

### Neuronal Correlates

The neuroimaging correlates of SoA have been reviewed by David et al. (2008) and Haggard (2017). Following David et al. (2008), the identified brain areas may be distinguished into two groups. The first group comprises brain regions known to be involved in the motor system, such as the supplementary motor areas, the ventral PMC and the cerebellum. The second group encompasses heteromodal association cortices, including the posterior parietal cortex, the dorsolateral prefrontal cortex, the posterior segment of the superior temporal sulcus and the insula. As David et al. (2008) point out, how all these different brain regions contribute to the emergence of SoA, remains speculative. Partly, this may be due to a methodological weakness in many SoA studies in that they have used experimental manipulations that do not carefully enough separate those neuronal processes underlying (non-conscious) action control from those subserving the actual subjective SoA experience (Haggard, 2017). Nonetheless, a functional involvement of the sensorimotor areas and parietal cortex appear plausible in face of the CM (Tsakiris et al., 2007b; Desmurget and Sirigu, 2009). Likewise, an involvement of the (anterior) insula appears comprehensible, given its general assumed involvement in bodily self-awareness (Baier and Karnath, 2008; Craig, 2009; Karnath and Baier, 2010).

## Interplay Between SoA and SoO

In the last two sections, SoO and SoA have been presented in isolation. In many of our everyday life situations, we, however, experience both SoO and SoA together. For instance, if I lift my leg, then I experience not only SoO for my leg, but also SoA for my leg movement. This raises the question of whether SoO and SoA just arbitrarily co-occur in our experiences, or whether they systematically interact with each other. To follow up this question, a few experimental studies have been carried out, most of them relying on “active” RHI designs (Tsakiris et al., 2006, 2010b; Dummer et al., 2009; Sanchez-Vives et al., 2010; Zeller et al., 2011; Kalckert and Ehrsson, 2014a,b; Braun et al., 2014). What these RHI designs have in common is that the RHI is not induced by visuotactile stimulation, but by synchronous movement between the artificial and participant’s real hand.

Among the first active RHI studies is Dummer et al.’s (2009) study. Focusing on whole hand movements, Dummer et al. (2009) connected the participant’s real hand with the artificial hand by a brace, such that whenever the participants moved their own hand forth or back, or the experimenter moved the brace forth and back, the participant’s real hand and artificial hand moved correspondingly. This allowed the authors to investigate whether an RHI can also be induced by visuomotor rather than visuotactile synchrony. And if yes, whether the RHI is stronger under active or under passive hand movements. As regards the first research question, Dummer et al. (2009) indeed found an RHI inducibility for their movement conditions. The reported SoO levels, however, were clearly weaker compared to their classical visuotactile RHI condition. As regards the second research question, Dummer et al. (2009) found some trendwise indications for stronger SoO under active than passive movements. Since the only difference between both movement conditions was whether the participants voluntarily self-generated or only passively underwent the movements, this finding suggests that voluntary action, or at least some component of it (e.g., efferent motor signals or SoA), have a promoting effect onto our SoO experience.

Also Walsh et al. (2011) investigated the inducibility of SoO under active and passive movements. In contrast to Dummer et al. (2009), however, Walsh et al. (2011) focused on index finger movements rather than whole hand movements and they anesthetized the index finger used for the movements. This allowed them to selectively block the tactile, but not proprioceptive sensory information coming from the moving index finger. What Walsh et al. (2011) found was that even in the complete absence of tactile sensory information, an illusory SoO over a fake finger can be induced, as long as the fake finger moves in spatiotemporal synchrony to the participant’s own finger. Moreover, in contrast to Dummer et al. (2009) and other studies (Kalckert and Ehrsson, 2012; Braun et al., 2014), Walsh et al. (2011) found no evidence that voluntary movements induce stronger illusory SoO than passive movements. Walsh et al. (2011) therefore suggested that voluntary action is in fact not crucial for inducing SoO.

**FIGURE 4:**
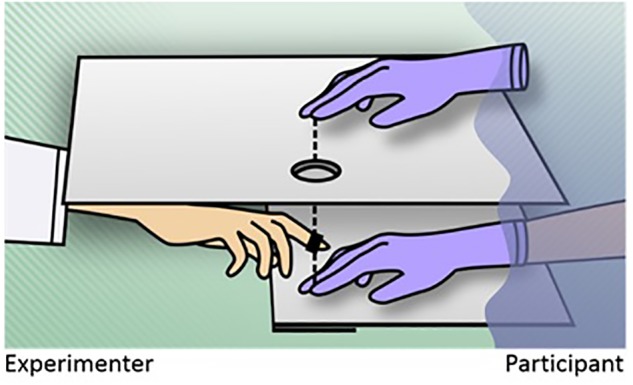
The active rubber hand illusion. The participant’s index finger and artificial index finger are connected via a small rod (dashed line). As a result, whenever the participant moves his or her own index finger up or down, or alternatively the rod is moved up or down by the experimenter, the artificial hand’s index finger moves correspondingly. The occurrence of SoO and SoA can be systematically investigated by varying the mode of agent (i.e., whether the artificial finger movements are self-generated or generated by the experimenter) and by the positioning of the artificial hand (Braun et al., 2014; Kalckert and Ehrsson, 2014a,b).

While the two just presented studies only focused onto the functional role of movement for SoO, a systematic investigation of the interplay between SoO and SoA in either direction was conducted by Kalckert and Ehrsson (2012). Presumably inspired by Walsh et al. (2011), they developed a vertical RHI variant where the artificial hand’s index finger is movable and connected to the real hand’s index finger by a tiny rod that goes through the upper plate (see **Figure 4**). As a result, whenever the participants move their own index finger, or whenever the rod is moved by the experimenter, the artificial hand index finger moves correspondingly. Using this general setup, the mechanisms contributing to SoO and SoA could be systematically investigated by, inter alia, varying the mode of agent (i.e., whether the artificial finger movements were self-generated or experimenter-generated) and the position of the artificial hand (i.e., whether the artificial hand was placed in anatomical alignment or misalignment to the participant’s real hand). One important result was that SoO and SoA were both inducible by this paradigm. Another important finding was that SoO and SoA could be experimentally double-dissociated, but if the experimental conditions allowed their concomitant emergence, they also mutually strengthened each other. In other words, SoO mainly depended on anatomical hand congruency, but, to a lesser extent, also on whether the artificial finger movements were self-caused, or not. SoA, in turn, mainly depended on self-causation of the artificial finger movements, but, to a minor extent, also on whether the artificial hand was anatomically aligned. These two main findings were replicated by another study from Braun et al. (2014) that used quite similar RHI and factorial designs as the ones used in Kalckert’s and Ehrsson’s (2012) study.

If SoO and SoA promote each other, this raises the question of why and how they do so. As regards a possible promoting influence of SoA on SoO, a possible working hypothesis could be that voluntary action is an important source of information for self-recognition (Van Den Bos and Jeannerod, 2002; Synofzik et al., 2008b). That is, by moving the body, the brain can test its predictions about which sensory events reflect the own body and which ones do not (Synofzik et al., 2008b). Moving the body may thus sharpen one’s own bodily boundaries and induce a more vivid SoO experience. As regards a possible promoting influence of SoO on SoA, it should be recalled here that although our actions often range beyond our bodily boundaries, they always originate in our body (Wong, 2010). Hence, it appears logical if our brain attributes higher certainty levels of authorship to our immediate body actions than to our less foreseeable effects on the world.

In sum, there is still some variability in results, especially concerning the question whether voluntary action (SoA) has a promoting effect onto SoO (Dummer et al., 2009; Kalckert and Ehrsson, 2012; Braun et al., 2016, 2014), or not (Walsh et al., 2011). The overall picture, however, indicates that although both experiences can partially double dissociate (Kalckert and Ehrsson, 2012; Braun et al., 2014), if they co-occur in experience, they may strengthen each other (Dummer et al., 2009; Kalckert and Ehrsson, 2012; Braun et al., 2014, 2016).

## Toward a Unified Theory of Self-Awareness

Astonishingly, although the just presented theoretical arguments and empirical findings speak for a strong interaction between SoO and SoA, most existing neurocognitive theories only offer an explanation for either SoO or SoA, but not for both phenomena together. A few predictive coding (PC) accounts, have, however, recently been put forward, that seek to explain self-awareness in general, including SoO and SoA (e.g., Hohwy, 2007; Limanowski and Blankenburg, 2013; Apps and Tsakiris, 2014; Tsakiris, 2016).

PC accounts regard the brain as a Bayesian-like prediction machine that tries to infer the hidden causes of its sensory input (Friston, 2010, 2012). The idea is that the brain constructs hierarchical generative models about the hidden causes of its sensory input and continually attempts to minimize its models’ prediction errors on each hierarchical level. To this end, the brain derives sensory predictions from its models, and then tests these predictions against its incoming sensory input (Friston, 2010; Clark, 2013). If there is a match between the predicted and actual sensory input, the respecting model is confirmed. If there is a mismatch, prediction error occurs and the model needs to be updated (Friston, 2010, 2012; Friston et al., 2013).

While PC accounts are not really new, dating back at least to Helmholtz (1821–1894), what is novel is their currently considered relevance for explaining self-awareness. In a nutshell, the common idea of these accounts is that not only perception, but also self-awareness, arises by PC (Limanowski and Blankenburg, 2013; Seth, 2013; Apps and Tsakiris, 2014; Tsakiris, 2016). That is, the brain not only generates hierarchical generative models about the causal structures of the outer world, but also about the “the most likely to be ‘me”’ (Tsakiris, 2016, p. 8), and then it attempts to minimize its self-models‘ prediction errors. If this is the case, an interesting consequence would be that not only perception is probabilistic, but so too is self-awareness (Limanowski and Blankenburg, 2013). That is, the phenomenal content identified as mine is then nothing more than the most likely cause, given the current sensory input and some top–down priors (Seth, 2013; Tsakiris, 2016). This assumption nicely fits with the known – experimental and clinical – malleability of SoO and SoA.

An intuition about how SoO might depend on minimizing a generative model’s prediction error, can for instance be given by using the RHI as an example (Kilteni et al., 2015; Samad et al., 2015). To infer whether its current multisensory input has one common cause (i.e., only one self-owned hand) or two causes (i.e., one self-owned hand and an additional artificial hand), the brain compares the probabilities for whether its unimodal limb sensations have common or independent causes and thereby takes into account the similarity of the sensations and the prior probability of a common cause. If the estimated probability for a common cause is higher than the estimated probability for two different causes, the brain fuses the two disparate limb sensations together and an RHI occurs. That is, the brain explains its prediction error away by updating the generative model. If the evidence for two separate causes prevails, however, the generative model is not updated and the two disparate limb sensations remain perceptually apart, thus no RHI occurs.

Illusory SoO during the RHI may thus come about by updating the generative model – a mechanism known as perceptual inference. As Friston (2010) and Friston et al. (2013), however, emphasize, perceptual inference is not the only way how the brain may reduce its prediction errors. Its alternative is to perform an action, to bring about a new sensory state in line with the model’s predictions – a mechanism known as active inference (Friston, 2010). That is, if there is uncertainty whether the current model’s predictions are right, the brain may conduct a “reality check” by conducting an action (e.g., moving the real right hand during a right-sided RHI), whose sensory consequences are potentially easier to predict than the current sensory state (Hohwy, 2007; Limanowski and Blankenburg, 2013). If the initiated action then leads to a predicted sensory outcome, SoA arises and the current self-model can be maintained. If the initiated action does not lead to the predicted sensory outcome, no SoA arises and the self-model has to be adapted to the new sensory state.

In sum, although PC accounts of self-awareness are still in an early developmental stage, they may provide an interesting explanatory alternative to the existing SoO and SoA accounts.

## Clinical Disruptions and Therapeutic Relevance of SoO and SoA

Under normal healthy conditions we typically have no problems in identifying our own body parts, feelings, thoughts and actions. Various clinical conditions, however, exist under which these ordinary SoO and/or SoA experiences are compromised. As illustrated in the following, some of these disruptions are remarkably specific and may be restricted to one specific body-part, while other SoO/SoA disruptions affect the global self.

### Limb-Specific Disruptions of SoO and SoA

Limb-ownership can become disrupted in many different ways (see **Table 1**). Whereas in some conditions, limb-ownership ceases to exist (asomatognosia, somatoparaphrenia; Feinberg et al., 2010) or gets a strong aversive connotation (misoplegia; Loetscher et al., 2006), in other conditions it persists, but it’s phenomenal content no longer relates to any physical counterpart (phantom limb; Hill, 1999). Similarly, it may happen that an additional phenomenal limb becomes instantiated in the absence of an additional physical limb (supernumerary limb; Cipriani et al., 2011).

**Table 1:** Limb-specific disruptions of SoO and SoA.

Clinical condition	Clinical description	Reference
Asomatognosia	Non-recognition and/or denial of ownership of one’s own limb.	Feinberg et al., 2010
Somatoparaphrenia	Severe subtype of asomatognosia, in which patients also display delusional misidentification (e.g., misattribution of limb to another person) and/or confabulation (e.g., personification).	–
Supernumerary limb	Illusory experience of the presence of an additional limb.	Cipriani et al., 2011
Misoplegia	Morbid dislike or hatred of a limb.	Loetscher et al., 2006
Phantom limb	Illusory feeling that an amputated or missing limb is still present and can be moved.	Hill, 1999; Flor, 2002
Anarchic Hand Syndrome	Experience of one’s own limb actions as alien.	Feinberg et al., 1992; Sarva et al., 2014
Anosognosia for Hemiparesis	Unawareness of one’s own contralesional motor deficits.	Pia et al., 2004


Also SoA may become selectively disrupted for a specific limb or body part. Whereas patients with anarchic hand syndrome (sometimes also referred to as alien-hand syndrome) for instance experience their own purposeful limb actions as alien and non-intended (Sarva et al., 2014), i.e., loose their SoA for their affected limb, patients with anosognosia for hemiparesis still report illusory SoA for their now plegic body parts (Pia et al., 2004). Moreover, some phantom limb patients report an illusory SoA for their absent phantom limb (Moseley and Brugger, 2009).

### Other Domain-Restricted Disruptions of SoO and SoA

Circumscribed SoO/SoA-disruptions may also occur toward one‘s own thought processes (see **Table 2**). Patients suffering from psychosis, for instance, often experience some of their own thoughts as alien and non-self-caused (made thoughts). As a consequence, they often deny SoO and/or SoA over their thoughts or even misattribute them to another person or metaphysical force (thought insertion) (Mellor, 1970). Patients with obsessive-compulsory disorder, in turn, often report intrusive thoughts that they still identify as mine but that they don’t want to have and whose emergence they can’t control (Markarian et al., 2010).

**Table 2:** Thought- and emotion related disruptions of SoO and SoA.

Clinical condition	Description	Literature
Made thoughts	Experience of one’s own thoughts as alien.	Gunn, 2016
Thought insertion	Delusional belief that one’s own thoughts belong to someone else.	
Made feelings	Experience of one’s own feelings as alien.	Mellor, 1970
Obsessive thoughts	Intrusive, unwanted thoughts that persist despite efforts to suppress, resist, or ignore them.	Markarian et al., 2010
Grandiosity delusions	Fantastical belief about being omnipotent and having inflated worth, power, knowledge or a special identity.	Knowles et al., 2011


Likewise, several affective symptoms exist where patients over- or underestimate their causal influence on the world (see **Table 2**) (Gentsch and Synofzik, 2014). For instance, whereas manic patients often experience an inappropriately strong SoA and sometimes even feel omnipotent (grandiosity delusions; Knowles et al., 2011), depressive patients tend to overestimate their agentic contributions to negative action outcomes and to underestimate their agentic contributions to positive action outcomes (Gentsch and Synofzik, 2014).

Moreover, emotion-related SoO distortions also exist. Patients suffering from psychosis, for instance, sometimes describe their own feelings and emotions as alien or as belonging to another person or power (made feelings; Mellor, 1970).

### Disruptions of Body-Ownership

Besides limb-related SoO distortions, several clinical symptoms exist where also body-ownership becomes disrupted (see **Table 3**). Although rare in occurrence, several autoscopic phenomena have been documented over the last decades (Blanke et al., 2004; Zamboni et al., 2005; Anzellotti et al., 2011). Under these severe clinical conditions, multiple, competing bodily self-representations become instantiated and cannot be integrated to one unified subject of experience anymore. Following Blanke and Metzinger’s (2009) classificatory attempt, at least three different autoscopic phenomena may be distinguished: First, an autoscopic hallucination where an illusory duplicate of the own body is seen in the extracorporeal space, but the patient still spatially situates him- or herself at the position of his or her physical body and experiences this physical body as mine. A weaker, kinaesthetic variant of this hallucination is the shadow person phenomenon. Here, the patients have the diffuse kinaesthetic impression that another person sits closely beneath their back (Arzy et al., 2006). Second, an out-of-body experience (OBE) where the patients experientially float outside their physical body and see themselves from outside. That is, self-location, SoO and 1PP are experienced for the illusory body and not for the own physical body. And third, heautoscopy where again a second illusory body becomes hallucinated, but here the person either identifies him- or herself with the physical body, illusory body or with both bodies at the same time. That is, heautoscopy is an intermediate form between an autoscopic hallucination and OBE where SoO, self-location and 1PP unstably switch back and forth between both competing body-representations.

**Table 3:** Disruptions of body-ownership.

Clinical condition	Description	Reference
Shadow person	Feeling of presence of a person nearby.	Arzy et al., 2006
Autoscopic hallucination	Illusory experience of a duplicate of one’s own body in extrapersonal space. 1PP and self-identification remain in the “physical” body.	Blanke et al., 2004; Metzinger, 2007b; Blanke and Metzinger, 2009; Blanke, 2012
Out-of-body experiences	Illusory experience of floating outside one’s own body. 1PP and self-identification are situated in the “illusory” body.	
Heautoscopy	Illusory experience of a duplicate of one’s own body in extrapersonal space. 1PP and self-identification are either in the physical body, “illusory” body or in both bodies situated.	


### Global Disruptions of SoO and SoA

Besides the just mentioned domain-restricted SoO/SoA-disturbances, there are also some SoO/SoA disturbances that affect the global self (see **Table 4**). Pathologically weakened forms of SoO and SoA can for instance be observed under dissociation (World Health Organization [WHO], 1992), depersonalization and in Cotard’s syndrome. Whereas under dissociation and depersonalization, the patient still possesses an intact, non-psychotic self-representation, but experiences this representation as detached, alien or unreal (Hunter et al., 2004; Ataria, 2015), in Cotard’s syndrome the patient does not identify with his self-representation anymore, but instead completely denies his or her own ontological existence (Debruyne et al., 2011). A third example, exclusively relating to SoA, would be delusions of control (Frith, 2004) where the patient is convinced that someone or something else (e.g., a supernatural power) controls his or her whole actions.

**Table 4:** Global disruptions of SoO and SoA.

Clinical condition	Description	Literature
Delusion of control	Delusional belief that someone or something else controls all of one’s own actions.	Frith, 2004
Depersonalization	Experiences of unreality, detachment, or being an outside observer with respect to one’s thoughts, sensations, actions or feelings.	Lambert et al., 2002; Hunter et al., 2004
Dissociation	“Partial or complete loss of the normal integration between memories of the past, awareness of identity and immediate sensations, and control of bodily movements.”	World Health Organization [WHO], 1992
Cotard’s syndrome	Delusional belief that one is dead or no longer exists.	Debruyne et al., 2011


In sum, many clinical symptoms can be found where either SoO, SoA or both phenomenal experiences are disrupted. This highlights the clinical relevance of both phenomenal experiences and motivates their further clinical and experimental investigation.

### Therapeutic Relevance of SoO and SoA

A careful consideration of SoO and SoA may also be of interest for improving therapeutic interventions. As regards SoA, it may be argued that experiencing SoA is crucial for almost every therapeutic approach whose mechanism of action is based on an active patient contribution. Our reasoning here is that self-efficacy (i.e., the confidence in being able to achieve intended goals; Bandura, 1977) is an important requirement for any willingness to take action and that self-efficacy comes about by repeatedly making positive SoA experiences. Implementing a therapeutic intervention facilitating SoA may thus be a promising strategy to motivate patients to act, and to uphold their actions. This reasoning may apply in particular to depression syndromes, where a loss of self-efficacy is thought to be a crucial upholding factor of this disease (Ehrenberg et al., 1991). Practically, a SoA boost could be for instance realized by making the patients more aware of their actions and immediate impacts onto the world.

As regards SoO, its therapeutic potential comes into play in interventions that potentially allow for embodiment. One example is the design of neuroprosthetic devices, such as myoelectric prosthetic arms (Geethanjali, 2016), cochlear implants (Zeng et al., 2008) or powered exoskeletons (Miller et al., 2016). Defining embodiment as a special form of neuronal information processing, in which a perceptual object is processed in the same way as if it was part of the own body (De Vignemont, 2011), it may be argued that an *embodiable* prosthetic device is an intuitive and phenomenally transparent device. On the phenomenal level, a strongly perceived SoO for this device would thereby be one important phenomenal target property to be achieved.

Another intervention with a potential for embodiment is neurofeedback-guided motor imagery training (NF-MIT). In this intervention, paretic stroke patients receive online neurofeedback about their brain activity whilst conducting a motor imagery task (Zich et al., 2015). The rationale behind NF-MIT is to feed back to the patients when they are performing well, that is, show a beneficial neuronal activation pattern in respect to motor recovery, and when not (Sitaram et al., 2016). In most studies the neurofeedback signal is rather abstract and not intuitively coupled to the MI act performed (Lotte et al., 2013). This, however, does not need to be the case. Alternatively, an *embodiable* neurofeedback signal could be provided that closely matches the mental act performed, in both time and space. Practically, this can be for instance realized by encoding the neurofeedback signal in the movements of an anthropomorphic robotic hand that is placed in a RHI-like setting and therefore can be experienced as being part of one’s own body (Braun et al., 2016). Likewise, first VHI implementations have been presented that also allow for an embodiable neurofeedback signal (Perez-Marcos et al., 2009; Alimardani et al., 2013; Ono et al., 2013; Pichiorri et al., 2015).

A third approach where artificially enabling SoO appears therapeutically promising is virtual reality immersion therapy (VRIT) (for reviews and critical discussions, see Riva, 2005; Sanchez-Vives and Slater, 2005; Freeman et al., 2017). Relying on VR technology, VRIT generates interactive virtual environments for therapeutic purposes. One concrete VRIT application is for instance VR exposure therapy where phobic patients immerse into a fearful virtual environment and thereby learn to cope with their fears (Meyerbröker and Emmelkamp, 2010). One phenomenal target property that shall be realized by VR interventions is perceptual presence, that is, the experience of being situated in the virtual environment (Sanchez-Vives and Slater, 2005; Freeman et al., 2017). It may be argued here that, practically, this requires that, besides other crucial factors (for critical discussions, see Sanchez-Vives and Slater, 2005; Slater, 2009), the avatar representing oneself in the virtual environment has to be implemented in such a way that illusory SoO can be experienced for the avatar. The OBI and VHI research presented above (see Experimental Investigation) is therefore not only neurophilosophically interesting, but also potentially helpful in developing new VRIT interventions. In summary, several therapeutic interventions could benefit from a better consideration of SoO and SoA.

## Conclusion

This paper reviewed the neurocognitive underpinnings, interplay, clinical disruptions and therapeutic potential of SoO and SoA. While the presented clinical disruptions and bodily illusions demonstrate an astonishing malleability in what we identify as mine and as our agentive contribution to the world, the reviewed experimental paradigms and neurocognitive theories illustrate that this malleability is not arbitrary, but empirically tractable and predictable. We argue that modern therapeutic interventions may benefit from a more careful consideration of SoO and SoA.

## Author Contributions

NB wrote major parts of the manuscript. SD, NS, EB, PS, HM, and AP contributed to, reviewed, and edited the manuscript.

## Conflict of Interest Statement

HM received speaker’s compensation from HM received speaker’s compensation from LivaNova within the last year. The other authors declare that the research was conducted in the absence of any commercial or financial relationships that could be construed as a potential conflict of interest.
